# Commemorating The Nobel Prize in Chemistry 2023 for the Discovery and Synthesis of Quantum Dots

**DOI:** 10.1021/acscentsci.3c01296

**Published:** 2023-11-02

**Authors:** Kui Yu, Kirk S. Schanze

In this editorial, we pay tribute to The Nobel Prize in Chemistry 2023 awarded “for the discovery and synthesis of quantum dots”. Quantum dots (QDs) are comprised of semiconductors, whose electronic properties are relatively unaffected by their sample size when in their bulk state. When the size of semiconducting materials becomes smaller and smaller approaching that of a molecule or an atom, their electronic properties and thus optical properties transform from relatively size-independent to size-dependent. This is conceivable. For atoms and molecules, their intrinsic electronic properties are governed by their distinctive sizes and shapes. When the size of semiconductor nanomaterials determines their electronic properties, and they are approximately spherical in shape, they are called QDs. Nowadays QDs play a key role in nanotechnology and nanoscience.

QDs are zero-dimensional (0D) semiconductors with sizes in the nanometer range. Under optical radiation, excitons are produced which are Coulombically bound electron–hole pairs. Usually, an exciton has a Bohr diameter that is larger than or similar to the QD size; thus, the exciton is three-dimensionally (3D) confined. Consequently, QD size matters. The smaller the QD, the stronger the confinement. When the excited QD relaxes by radiative decay, the luminescence wavelength becomes shorter as the QD size decreases. To study quantum size effects, both the size and size distribution of the semiconductor nanoparticles are important parameters. Ideally, the precision of particle size can be controlled on the atomic level. About 40 years ago, it was barely possible to have man-made colloids whose electronic properties could be systematically controlled to be size-dependent.

We now extend our congratulations to the three award winners, Moungi G. Bawendi at Massachusetts Institute of Technology (MIT, USA), Louis E. Brus at Columbia University (USA), and Alexei I. Ekimov at Nanocrystals Technology Inc. (USA) They succeeded in producing reasonably homogeneous semiconductor nanoparticles such that changes in their size-dependent electronic and optical properties could be distinguished. The synthesis and characterization of these small particles mark a significant milestone, signifying a groundbreaking development in the field of nanotechnology that greatly enhances our daily lives. The three winners are respected pioneers of nanotechnology and nanoscience.

About 40 years ago, **Alexei I. Ekimov** began studying semiconductor-doped glasses at the S. I. Vavilov State Optical Institute (Russia).^1^ The manufacturing technology of glass that contains colloidal particles goes back more than a thousand years, and colorful glasses are present in the windows of medieval churches. The fabrication protocol in old alchemy books can be summarized as, put this and that chemical compounds into melting glass (at temperature *T*_1_), let glass cool down to this temperature (*T*_2_), and then heat it at that temperature (*T*_3_) for this duration of time. The glass can thus be beautifully colored through the inclusion of small particles of metals, metal oxides, and semiconductors. The particle-activated glass attracted Alexei’s attention, and he decided to study the underlying cause of the colors’ appearance.

Alexei had the great idea to focus his investigation on glasses that contained only one type of particle, and to study the chemical composition, structure, and growth of the particles inside the glass. Four types of particles were studied—CuCl, CuBr, CdS, and CdSe. Alexei manipulated experimental parameters such as the sample annealing temperature (*T*_3_) and first succeeded in managing the color of glass that was activated by CuCl particles. Alexei demonstrated that when a sample was annealed at a relatively low temperature, its optical absorption peaked at a relatively short wavelength, and the CuCl particle size was relatively small. The color of the glass came from the CuCl particle inside the glass, and the size of the particle (in the range of 2 to 30 nm) plays an important role in the color’s appearance.

As a physicist, Alexei quickly realized that the size-dependent optical properties observed was a size-dependent quantum effect.^2^ The concept of quantum size effects had already been developed by physicists, and it could now be tested by various small man-made particles in the real world.^3^ Also, Alexei tried to decrease the size distribution of the particles, the fabrication of which involved fast nucleation at a relatively high temperature (*T*_1_) followed by speedy cooling of the glass for relatively slow growth of particles. This method has an apparent likeness of the previous work of LaMer and Dinegar in 1950,^4^ and the later hot-injection approach reported by Moungi G. Bawendi and co-workers in 1993.^5^

Independently and almost simultaneously at the beginning of the 1980s, **Louis E. Brus** at Bell Laboratories (New Jersey, USA) was also studying colloidal semiconductor particles of II–VI metal chalcogenide (ME) that were dispersed in aqueous environments. In late 1982, Louis accidentally noticed that the optical absorption of an aged sample of CdS colloids in aqueous solution changed on occasion. Louis thought it through and assumed perceptively that the change was due to a quantum size effect. In order to test this assumption, Louis decided to control the CdS size via precursor concentration and growth temperature (*T*_3_). Upon the manipulation of the experimental parameters, Louis was able to systematically study freshly prepared and aged CdS colloids.^6^ The former particles were smaller (4.5 nm), and the latter ones were larger due to Ostwald ripening (12.5 nm), with the absorption peaks red-shifting to longer wavelengths accordingly. Such a spectral change is in agreement with the size change, which is due to the quantum size effect. Concurrently, other researchers including Arnim Henglein in Germany were also making efforts in the study of semiconductor colloidal dispersions.^7^ This could be related to the research interest during the 1970s, which was quite significant in the field of semiconductor photoelectrochemistry.

Motivated by the experimental observation, Louis developed the famous “Brus formula” that describes the relationship between the size and bandgap of colloidal semiconductor QDs.^8,9^ This mathematical expression quantitatively contains the kinetic energy (that is related to effective masses of electrons and holes) and the potential energy (that is related to dielectric polarization). For the potential energy, Louis used classical electrostatics together with the idea that the Coulomb interaction between electrons holes is screened in small colloidal crystallites. On a side note, it was in 1986 that the term “quantum dot” was introduced by Mark Reed for small particles that are 0D semiconductors;^10^ the terminology sensibly follows the existing one for 2D quantum wells and 1D quantum wires.

While the theoretical understanding of the size-dependent bandgap of particles was developing,^11,12^ Louis was also actively seeking better methods for particle synthesis. In 1986 A. Paul Alivisatos joined Louis as a postdoctoral fellow. The wet-chemistry synthesis was labor intensive, and the three scholars, Louis, Paul, and Michael Steigerwald (a synthetic chemist) worked side-by-side on a daily basis. Various synthetic ideas were tried, including performing a reaction in an inert environment. Accordingly, techniques used by organometallic chemists were implemented, such as the use of a Schlenk vacuum line and a glovebox. For the size control of CdSe particles, an inverse micelle approach was carried out in heptane at room temperature.^13^ Moreover, Louis and Michael and other researchers tried the idea of sequential growth and found out that the CdSe/ZnS core–shell particles displayed photoluminescence.^14^ This is an important step, which illustrates that a shell with a larger bandgap facilitates surface passivation of a core so that the photoluminescence from the core can be promoted and enhanced. The use of a large-bandgap shell to confine excitons of a small-bandgap core is now widely used to achieve better performance of QDs.

In 1988 **Moungi G. Bawendi** joined Louis as a postdoctoral fellow and advanced the synthesis further. Moungi attempted the use of chemicals that are Lewis bases, with the idea that the chemicals may interact with surface Cd atoms of particles via coordinate covalent bonds (to act as ligands) and thus provide colloidal stability to the particles. For CdSe particles from an inverse micelle approach, Moungi placed them in 4-ethylpyridine near 160 °C under argon. Surprisingly, the refluxed CdSe particles had a zinc blende X-ray diffraction pattern and displayed sharper optical absorption peaks. Better QDs were produced via post-treatment at a higher temperature following the inverse micelle approach at room temperature. In 1989, tri-*n*-butylphosphine (TBP) was used to replace 4-ethylpyridine, together with a higher temperature of 260 °C. The refluxed CdSe particles (with the size of ∼4.0 nm) had a wurtzite X-ray diffraction pattern and displayed photoluminescence.^15−17^ This represented an encouraging result pointing to future directions in QD synthesis. Meanwhile, Moungi noticed that TBP from a used bottle (that was not completely empty) was better than from a new bottle, regarding the size control and photoluminescence of CdSe particles produced. The oxidation of some of TBP in the old bottle to tri-*n*-butylphosphine oxide (TBPO) was indicated by NMR. Consequently, synthesis with the high-temperature refluxing approach was changed to the use of a mixture of commercial TBP (90%) and commercial TBPO (10%). However, the reproducibility of this approach was not satisfactory. This is because the impurity of a secondary phosphine in a commercial tertiary phosphine plays a critical role in the nucleation and growth of QDs.^18−20^

Moungi left Bell Laboratories in 1990 and joined the Department of Chemistry at MIT. Moungi continued his efforts to improve the synthesis of colloidal CdE QDs.^5^ The research team used commercial tri-*n*-octylphosphine oxide (TOPO) as a reaction medium that has an even higher boiling temperature. The compound that is unstable in air Cd(CH_3_)_2_ was applied as a Cd source. The team introduced a hot-injection approach to synthesize Cd-based QDs, instead of the refluxing approach with CdE powders formed in inverse micelles. They rapidly injected a room-temperature mixture in commercial tri-*n*-octylphosphine (TOP) of Cd(CH_3_)_2_ and SeTOP into TOPO at ∼300 °C (*T*_1_). Immediately, the temperature dropped to ∼180 °C (*T*_2_), and they set the growth temperature at ∼250 °C (*T*_3_). The swift injection results in a burst of nucleation followed by relatively slow growth at the lower temperature. In this way, the growth stage was effectively separated from nucleation, and improved results were obtained regarding the control of size and size distribution of particles. These particles are suitable for further processing. Accordingly, the hot-injection approach opened the door for QD-based applications, which covers a broad spectrum in various areas such as bioimaging and biolabeling, LED, and photovoltaics.^21^ It was later revealed that impurities of phosphonic acids in commercial TOPO play an important role in the formation of CdSe nanostructures with different shapes.^22^

The hot-injection approach encourages researchers all over the world to keep improving the synthesis of colloidal semiconductor QDs. For example, air-stable Cd-containing compounds such as CdO and Cd salts were introduced.^23−25^ A heating-up approach instead of the injection-based approach was developed, and commercial 1-octadecene (ODE with a boiling temperature of ∼315 °C) was used as a reaction medium instead of a mixture of commercial TOP and TOPO.^24,25^ The noninjection-based approach produced CdE (E = S, Se, and Te) QDs with similar sample quality, with regard to size distribution and optical properties. Nowadays, QD research is heavily invested in and directed toward QD-based applications.^21^ For the technology in the near future, it is anticipated that QDs may also play a role in flexible electronics, small-size sensors, optically pumped lasers, and encrypted quantum communication. The success of the various QD-based applications largely depends on nanoparticle synthesis with enhanced accuracy.

Despite the remarkable advances in the synthesis of QDs, to date the nanoscience community remains far from being able to consistently control the synthesis of colloidal semiconductor QDs at an atomically precise level. Meanwhile, we lack a precise understanding of the molecular-level processes through which colloidal QDs are formed from inorganic precursors. An in-depth fundamental understanding of the nucleation and growth of QDs should be able to provide guidelines for better design and synthesis. Thanks to the noninjection-based approach,^24,25^ a reaction can be readily stopped in the prenucleation stage,^26−28^ which is almost impossible for the injection-based approach.^5,23^ Prenucleation clusters that are relatively transparent in optical absorption were trapped.^26−28^ They form via chemical self-assembly, that occurs prior to nucleation and growth of QDs.^26−29^ These prenucleation clusters can transform to magic-size clusters (MSCs) and QDs.^26−28^ Thus, a multistep nonclassical model should be taken into account to understand the nucleation and growth of colloidal QDs, instead of the one-step classical LaMer model.^4^ It is highly possible that MSCs and QDs are regulated differently in relation to quantum size effects because shape comes into play for the former.^28,30,31^ On a side note, CdSe MSCs (with a size of ∼1.2 nm and optical absorption peaking at ∼415 nm) were produced in the hot-injection approach;^5^ the bandwidth of MSCs is narrower due to the almost complete absence of size distribution.

This Editorial introduces mainly the initial and original work carried out by the three award winners, with the objective to commemorate The Nobel Prize in Chemistry 2023 awarded “for the discovery and synthesis of quantum dots”. The 40-year-old field of QD research is still young. Building on the remarkable achievements of the three laureates, QD research will press forward with more confidence toward a basic appreciation of the chemistry that occurs in the prenucleation stage of QDs. In addition to QD-based applications, more momentum will be gained to comprehend the prenucleation stage of QDs, together with the formation and transformation of MSCs. We look forward to the day when the synthesis of both QDs and MSCs is no longer empirical science, but rather driven by mechanism-guided design and thorough comprehension for each synthetic receipt developed. It is our belief that colloidal semiconductor particles will shine more brightly to advance nanoscience and nanotechnology in the coming days.
